# Self-Assembled Monolayers of a Fluorinated Phosphonic Acid as a Protective Coating on Aluminum

**DOI:** 10.3390/molecules29030706

**Published:** 2024-02-03

**Authors:** Zhuoqi Duan, Zaixin Xie, Yongmao Hu, Jiawen Xu, Jun Ren, Yu Liu, Heng-Yong Nie

**Affiliations:** 1 College of Engineering, Dali University, Dali 671003, China; zhuoqiduan@126.com (Z.D.); yndlxzx@126.com (Z.X.); 2 Surface Science Western, The University of Western Ontario, London, ON N6G 0J3, Canada; jxu984@uwo.ca (J.X.); jren252@uwo.ca (J.R.); 3 School of Mechanical Engineering, Jiangnan University, Wuxi 214122, China; yuliu@jiangnan.edu.cn; 4 Department of Physics and Astronomy, The University of Western Ontario, London, ON N6A 3K7, Canada

**Keywords:** self-assembled monolayers (SAMs), fluorinated phosphonic acid (FPA), native oxide layer of aluminum, covalent bonding, surface roughening, hot water (HW) treatment, time-of-flight secondary ion mass spectrometry (ToF-SIMS), water contact angle (CA), surface and interface chemistry, surface morphology

## Abstract

Aluminum (Al) placed in hot water (HW) at 90 °C is roughened due to its reaction with water, forming Al hydroxide and Al oxide, as well as releasing hydrogen gas. The roughened surface is thus hydrophilic and possesses a hugely increased surface area, which can be useful in applications requiring hydrophilicity and increased surface area, such as atmospheric moisture harvesting. On the other hand, when using HW to roughen specified areas of an Al substrate, ways to protect the other areas from HW attacks are necessary. We demonstrated that self-assembled monolayers (SAMs) of a fluorinated phosphonic acid (FPA, CF_3_(CF_2_)_13_(CH_2_)_2_P(=O)(OH)_2_) derivatized on the native oxide of an Al film protected the underneath metal substrate from HW attack. The intact wettability and surface morphology of FPA-derivatized Al subjected to HW treatment were examined using contact angle measurement, and scanning electron microscopy and atomic force microscopy, respectively. Moreover, the surface and interface chemistry of FPA-derivatized Al before and after HW treatment were investigated by time-of-flight secondary ion mass spectrometry (ToF-SIMS), verifying that the FPA SAMs were intact upon HW treatment. The ToF-SIMS results therefore explained, on the molecular level, why HW treatment did not affect the underneath Al at all. FPA derivatization is thus expected to be developed as a patterning method for the formation of hydrophilic and hydrophobic areas on Al when combined with HW treatment.

## 1. Introduction

Control of the surface of materials is imperative to developing applications that rely on their surface-related functionalities. Self-assembled monolayers (SAMs) [1,2] of organo-phosphonic acids (PAs) [3] and organosilanes [4] formed on metal oxides have aroused enormous interest in interdisciplinary areas such as surface engineering [5,6], tribology [7], chemical sensors [8], corrosion protection [9] and electronics [10,11]. One of the applications of SAMs in molecular engineering is to control the wettability of a substrate. For example, SAMs of alkanethiols [2] on coinage metals and SAMs of alkylphosphonic acids [3,6,9,12] and alkylsilanes [4,5] on oxides are the most used systems to render the substrate hydrophobic. Due to the robust binding ability of organo-PAs onto various technologically important oxides including ITO, TiO_2_, Al_2_O_3_, ZrO_2_ and Fe_3_O_4_, studies towards understanding SAM formation mechanisms and developing applications have been carried out extensively [13,14,15,16,17]. It is commonly regarded that there are four binding modes for organo-PAs on metal oxides, namely, mono-, bi- and tridentate, as well as bidentate with electrostatic configurations [16]. The techniques used to investigate the reaction between the organo-PA headgroup (i.e., the anchoring group) and the substrate include infrared reflection absorption spectroscopy [15], X-ray photoelectron spectroscopy [6,14], density functional theory [14,16] and time-of-flight secondary ion mass spectrometry (ToF-ISMS) [18]. Because most SAMs are a couple of nanometers in thickness [19,20], accessing their surface and interface chemistry requires a surface-sensitive analytical technique. ToF-SIMS [21] perfectly fits this analysis requirement because it probes the outermost 1–3 nm of the surface and possesses superior chemical selectivity [22,23,24,25]. Some of the authors of the current article have applied ToF-SIMS to study the surface and interface chemistry of SAMs of octadecylphosphonic acid (OPA, CH_3_(CH_2_)_17_P(=O)(OH)_2_) and a fluorinated PA (FPA, CF_3_(CF_2_)_13_(CH_2_)_2_P(=O)(OH)_2_) [18,26]. Thermal stability SAMs of organo-PAs have also been studied both in ambient air [26] and in vacuum [14]. The degradation of SAMs is due to the decay of the perfluorocarbon backbone at temperatures of 250–300 °C [14,26], while the PA linkages with the substrate are not affected at temperatures as high as 650 °C, as determined by thermal stability studies in vacuum [14]. Therefore, the excellent thermal stability of organo-PA SAMs ensures their suitability in applications at temperatures that are less than 250 °C.

There have been extensive studies on enhancing hydrophobicity via surface roughening [26,27] or structural manipulations [28,29], which are necessary to achieve superhydrophobicity. It is well known that water contact angle (CA), a measure of wettability, on a rough hydrophobic surface will increase with increased surface roughness [30,31]. That is, a water droplet placed on a rough surface may penetrate into [30] or seal air within [31] the recessed areas of the surface, which corresponds to the Wenzel state and the Cassie–Baxter state, respectively. It has also been shown that there is a transitional state between these two states [27,28,29]. There has been increasing research interest in harvesting water from atmospheric moisture [32,33] by mimicking desert beetles (e.g., darkling beetles in the Namib Desert) that collect water droplets via condensation of fogs on their hardened shells [34,35,36]. This condensation phenomenon involves appropriately distributed hydrophilic and hydrophobic structures used to collect water droplets condensed from the moisture in the air. It has been shown that condensation occurs differently on hydrophobic and hydrophilic surfaces [37]. In other words, the wettability of a surface plays an important role in the moisture-harvesting mechanisms.

We have shown that immersing Al films (deposited on a Si wafer) in hot water (HW) at 90 °C resulted in roughening of the metal surface, on which the formation of SAMs of a fluorinated long-chain phosphonic acid rendered a superhydrophobic surface that is thermally stable up to 300 °C [26]. Therefore, the HW-roughened Al approach to rendering a hydrophilic surface with a huge surface area and a superhydrophobic surface when derivatized by FPA SAMs is expected to be applied in currently used atmospheric water-harvesting substrates [38,39,40], thanks to the enormously increased surface area achieved on a roughened Al surface.

To construct functionalities making use of both hydrophilicity and hydrophobicity, patterning of these two regions is required; in this article, we propose to use FPA SAMs as a mask to prevent the underneath Al substrate from HW attack. The kinetics of the formation of SAMs of organo-PAs has been thoroughly studied, showing that, at reasonable organo-PA concentrations, the completion of SAM formation on oxides takes minutes or tens of minutes [13,15]. The immersion time used in our experiments to prepare FPA SAMs was in the order of 10 h to ensure the completion of SAM formation. The formed FPA samples on the native oxide layer of Al before and after HW treatment were characterized by using water CA measurement, scanning electron microscopy (SEM) and atomic force microscopy (AFM), as well as ToF-SIMS to examine wettability, surface morphology and surface and interface chemistry, respectively. We demonstrate that the ability of FPA SAMs to resist HW attack enables the patterning of hydrophobic and hydrophilic areas over an Al substrate with HW-assisted roughening.

## 2. Results

**Figure 1:**
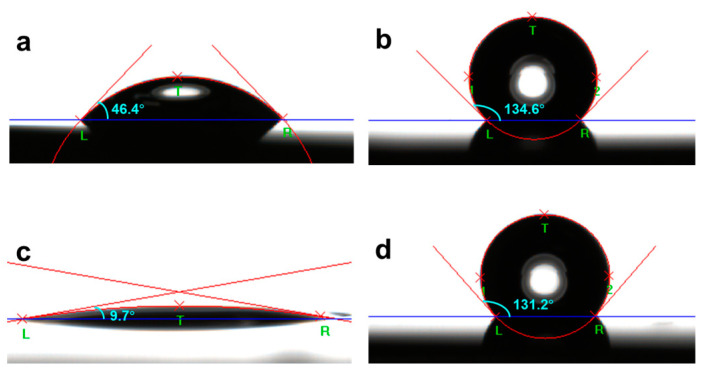
Photographs of a 3 µL water droplet placed on (**a**) cleaned Al, (**b**) FPA/Al, (**c**) HW-treated Al and (**d**) HW-treated FPA/Al. The Al film used for the experiment was 50 nm.

The Al substrate used was a thin Al film deposited on a Si substrate. The sample of FPA SAMs formed on the native oxide layer of the Al substrate is denoted as FPA/Al for simplicity. Shown in Figure 1a–d is an example of water CA measured on a cleaned Al film with a thickness of 50 nm and FPA/Al before and after HW treatment at 90 °C for 90 s. The static water CA on the cleaned Al was 46.4° (Figure 1a), which is typical for a substrate, such as Si wafer and metals, cleaned with organic solvents, suggesting the presence of adventitious hydrocarbons on the surface. Only when oxygen plasm [41] or UV ozone [42] treatment was used to clean the surface, which removes organic contamination from the surface, can CAs less than 10° be observed. Upon the formation of FPA SAMs on the cleaned Al, as shown in Figure 1b, the static water CA increased to 134.6°, reflecting that the surface is terminated by the perfluorocarbon tails of the FPA.

The HW treatment rendered the bare Al more hydrophilic, as evidenced by the CA decreasing to 9.7° (Figure 1c). Details on the roughness of the Al surface as a function of HW treatment time can be found elsewhere [26]. By contrast, the HW treatment resulted in a CA of 131.2° for the FPA-derivatized Al (Figure 1d), confirming that its hydrophobicity was intact. This experimental observation thus indicates that FPA SAMs protect the underneath Al from HW attack.

The static water CA data measured using a 3 µL droplet of ultrapure water are shown in Table 1, summarizing the averaged CAs with the standard deviations as evaluated from the data collected over five spots on each sample. The standard deviations of CAs are small, not exceeding 1.5° for the bare Al and FPA/Al. The standard deviations of CAs for the HW-treated bare Al and FPA/Al are as small as 0.5° and 0.1°, respectively.

**Figure 2:**
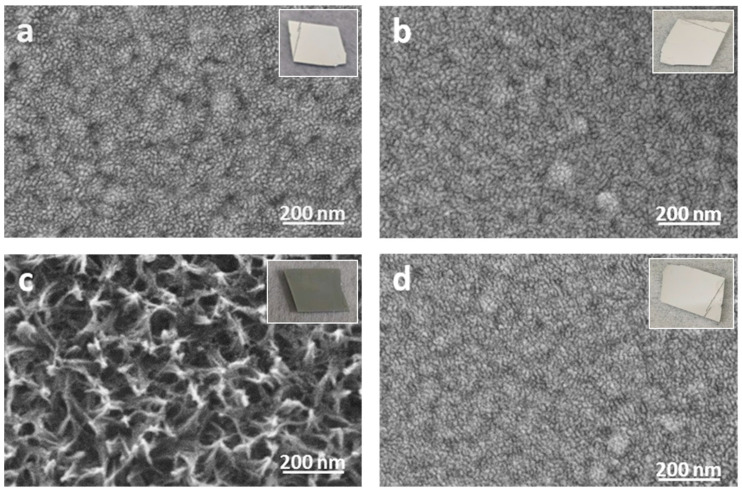
SEM images of (**a**) cleaned Al, (**b**) FPA/Al, (**c**) HW-treated Al and (**d**) HW-treated FPA/Al. The insert in each image is a photograph of the sample (in a size of 2 cm × 2 cm). The Al film used for the experiment was 50 nm.

Shown in Figure 2 are SEM images for the four samples described above, where the thickness of the Al film used for the experiment was 50 nm. The bare Al was roughened by HW treatment (Figure 2a vs. Figure 2c), which has been examined in detail in a previous publication [26]. By contrast, as shown in Figure 2b,d, there are no observable changes in surface morphology (as seen by SEM) of the FPA/Al before and after HW treatment. Therefore, the FPA SAMs protected the underneath Al substrate from HW attack. The insert in each SEM image is a photograph of the sample, with only the bare Al roughened by HW treatment appearing dark (Figure 2c), i.e., without having the metallic luster seen on the other three samples (Figure 2a,b,d). Therefore, a roughened Al surface by HW can be easily recognized visually.

**Figure 3:**
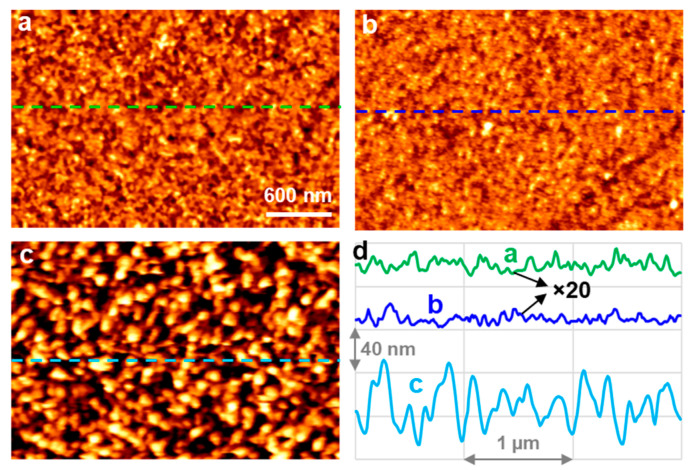
AFM images (scan area: 3 µm × 2 µm) of FPA/Al (**a**) before and (**b**) after HW treatment for 90 s. Shown in (**c**) is a bare Al after HW treatment for 90 s. A profile isolated from each image indicated by an inserted broken line is plotted in (**d**), where the height scale for those from a and b are multiplied by 20 for viewing purposes. The height scale for a and b is approximately 2 nm, while it is 110 nm for c. The Al film used for the experiment was 15 nm.

Except for the roughened Al (Figure 2c), there are no differences among the other three SEM images in Figure 2. To see if there are any detectable morphological differences between the FPA-derivatized Al before and after HW treatment, AFM was used to study their surface morphology. As shown in Figure 3a,b, the surface of the FPA/Al was intact upon HW treatment for 90 s. Specifically, the root mean square (RMS) roughness for both was around 0.2 nm, reconfirming that the FPA SAMs protected the underneath Al substrate. By contrast, the bare Al upon HW treatment for 90 s, as shown in Figure 3c, resulted in a drastic change in morphology, which corresponds to an 80-time increase in RMS roughness (i.e., 16 nm) in comparison with those of the FPA/Al, before and after HW treatment. Shown in Figure 3d are three profiles, each isolated from the images in Figure 3a–c, as indicated by the inserted broken line. The height scale (40 nm per division) in Figure 3d is for the profile isolated from Figure 3c. Because the profiles isolated from Figure 3a,b have a corrugation on the order of 1 nm, they are multiplied by 20 so that their height changes can be viewed.

We need to point out that the Al substrate used for the AFM and the following ToF-SIMS experiments was a 15 nm Al film deposited on a Si wafer, thus having a smoother surface in comparison with that of the 50 nm Al film used for the wettability (Figure 1 and Table 1) and SEM (Figure 2) experiments. We checked the roughness of the 50 nm Al film using AFM (Figure S1) and found that its RMS roughness was 1.7 nm, approximately 8 times that of the 15 nm Al film. Therefore, static water CAs were also measured for FPA SAMs formed on this smoother Al substrate for comparison. The averaged water CAs from the data collected over five spots on the FPA/Al sample before and after HW treatment at 90 °C for 90 s were 114.5° ± 1.7° and 114.4° ± 1.5°, respectively, with an example for each case shown in Figure S2. It is thus clear that the larger static water CA obtained on FPA SAMs formed on the rougher Al substrate (Figure 1b and Table 1) is due to the increased surface roughness [30,31]. For the smoother Al film, we also tested the resistance of SAMs of OPA to HW attack. The static water CAs before and after HW treatment were 112.6° ± 0.8° and 110.4° ± 0.6°, respectively, with an example for each case shown in Figure S3. Therefore, OPA can be an alternative to FPA for forming SAMs on the native oxide layer of Al to protect it from HW attacks.

**Figure 4:**
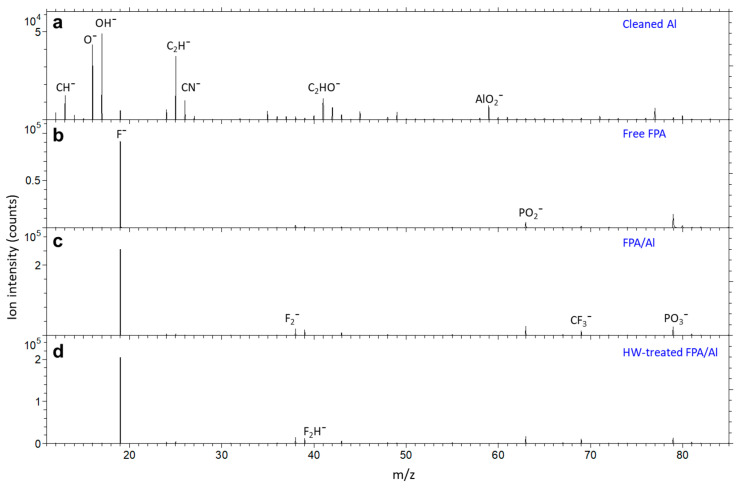
Negative secondary ion mass spectra for (**a**) cleaned Al, (**b**) free FPA molecule, and FPA/Al (**c**) before and (**d**) after HW treatment.

Having confirmed that FPA derivatization protected the underneath Al from HW attacking via water CA measurement and morphological evaluation using SEM and AFM, we further investigated the surface and interface chemistry of the FPA/Al before and after HW treatment using ToF-SIMS. Shown in Figure 4a–d are negative secondary ion mass spectra for a cleaned Al, a pure FPA molecule and FPA derivatized on Al before and after HW treatment, respectively. As shown in Figure 4a, the three most abundant ions from the cleaned Al are O¯ (nominal *m*/*z* 16), OH¯ (17) and C_2_H¯ (25). The carbon-containing ions CH¯ (12), C_2_H¯, CN¯ (26) and C_2_HO¯ (41) are due to surface contamination, including adventitious hydrocarbons [43]. A weak F¯ is detected, which is due to surface contamination of ionic fluorine. The most abundant negative ion characteristic of Al is AlO_2_¯ (59).

As shown in Figure 4b, the spectrum of free FPA molecules is dominated by F¯. This is also true for the FPA SAMs formed on an Al substrate before (Figure 4c) and after (Figure 4d) HW treatment. The other fluorine-containing ions shown in Figure 4b,c include F_2_¯ (38), F_2_H¯ (39) and CF_3_¯ (69), which are fragmented from the perfluorocarbon tails of FPA. The FPA headgroup is characterized by PO_2_¯ (63) and PO_3_¯ (79), which are detected from the free FPA molecules and the FPA SAMs before and after HW treatment.

**Figure 5:**
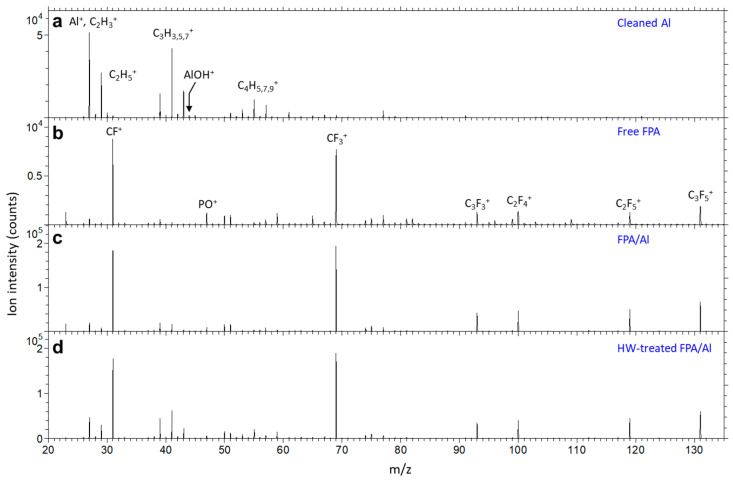
Positive secondary ion mass spectra for (**a**) cleaned Al, (**b**) free FPA molecule, and FPA/Al (**c**) before and (**d**) after HW treatment.

Shown in Figure 5a–d are positive secondary ion mass spectra for a cleaned Al substrate, a pure FPA molecule and FPA/Al before and after HW treatment, respectively. As shown in Figure 5a, Al^+^ (27) is the most abundant ion detected on the surface of the bare Al, and the other detected ions are hydrocarbon ions C_x_H_y_^+^ including C_2_H_3,5_^+^ (27, 29), C_3_H_3,5,7_^+^ (39, 41, 43) and C_4_H_5,7,9_^+^ (53, 55, 57), which are attributed to adventitious hydrocarbons [43]. Though not visible in Figure 5 (due to the intensity scale determined by the most abundant ions (i.e., Al^+^ for Figure 5a, CF^+^ and CF_3_^+^ for Figure 5b–d)), AlOH^+^ (43) is detected in the spectra shown in Figure 5a–d. Fluorocarbon ions in the spectra shown in Figure 5b–d are identical and are dominated by CF^+^ (31) and CF_3_^+^ (69). Other fluorocarbon ions include C_3_F_3_^+^ (93), C_2_F_4_^+^ (100), C_2_F_5_^+^ (119) and C_3_F_5_^+^ (131). These fluorocarbon ions are characteristic of a perfluorocarbon-containing material. Also noticed is the detection of PO^+^ (47) from the free FPA molecules and the FPA SAMs before and after HW treatment. This is the positive ion diagnostic of the headgroup of both free FPA molecules and FPA SAMs.

**Figure 6:**
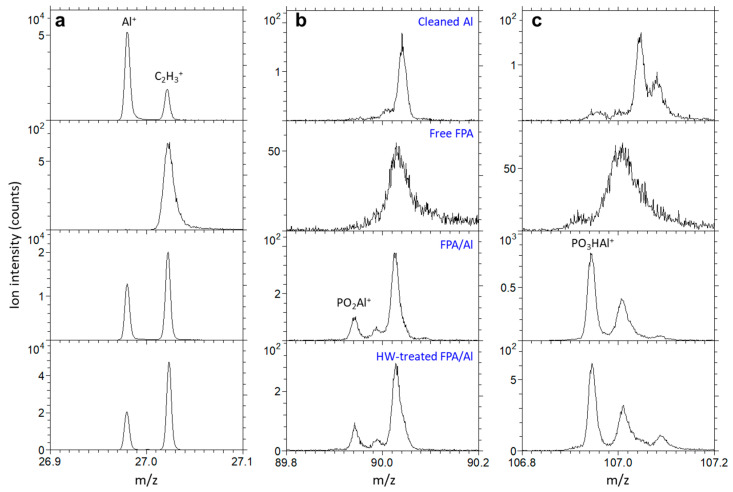
Positive secondary ion mass spectra at *m*/*z* 27 (**a**), 90 (**b**) and 107 (**c**) for cleaned Al, free FPA molecule and FPA/Al before and after HW treatment, showing the detection (or lack) of Al^+^, PO_2_Al^+^ and PO_3_HAl^+^, respectively.

Because both Al^+^ and C_2_H_3_^+^ have the same nominal *m*/*z*, their separation cannot be resolved in Figure 5a, where *m*/*z* ranges from 20 to 135. Detailed spectra at *m*/*z* 27 in Figure 6a show the detection of both Al^+^ and C_2_H_3_^+^ for the bare and the FPA/Al before and after HW treatment, as well as the lack of Al^+^ from the free FPA molecules.

Also shown in Figure 6b,c are spectra for PO_2_Al^+^ and PO_3_HAl^+^ at *m*/*z* 90 and 107, respectively, which reveal the covalent bonding between the FPA anchoring group and the underneath native oxide layer of the Al substrate because of the condensation reaction. The exact *m*/*z* values for the two ions assigned as PO_2_Al^+^ and PO_3_HAl^+^ are 89.9450 and 106.9420, respectively, when the spectra were calibrated using Al^+^, AlOH^+^ and PO^+^. The corresponding deviations, which are a measure of relative mass accuracy [44] on peak assignment, are −5.9 and −6.3 ppm against the theoretical *m*/*z* values of PO_2_Al^+^ (89.9446) and PO_3_HAl^+^ (106.9473), respectively. It is commonly accepted that deviations less than 55 ppm are required for correct ion assignment [45]. Therefore, such extremely small deviations (<10 ppm) in our case ensured the accuracy of the assignment of the two peaks to the two ions important in revealing the interface chemistry of FPA SAMs on the native oxide layer of Al. The lack of these two peaks in the spectra for the bare Al substrate and the free FPA molecules further supports the assignment of the two ions to PO_2_Al^+^ and PO_3_HAl^+^ for the FPA/Al before and after HW treatment.

## 3. Discussion

**Figure 7:**
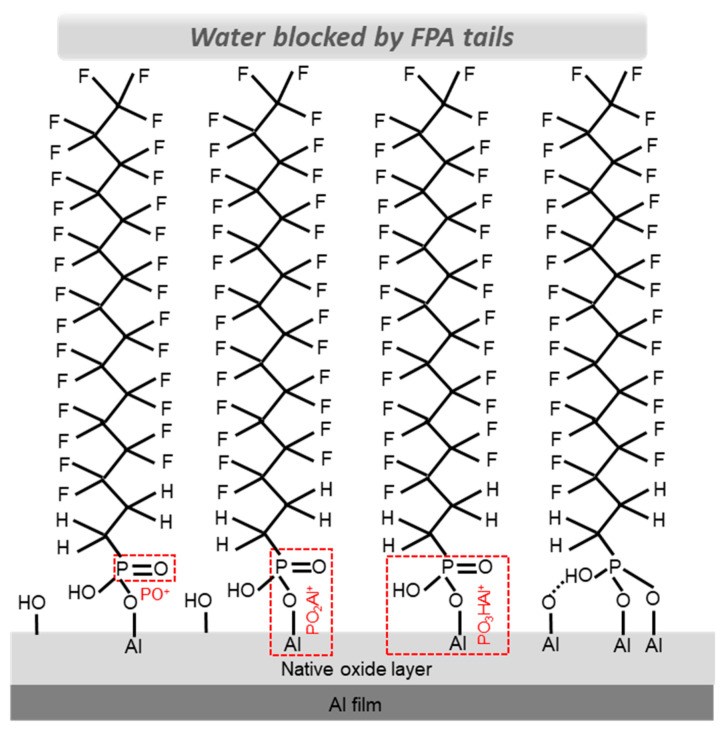
FPA SAMs formed on an Al substrate, with the formation of covalent bonding between the anchoring group and the native oxide layer of the Al film via condensation reaction. The binding modes shown are mono- and bidentate (with an additional electrostatic interaction) configurations. The positive secondary ions of PO^+^, PO_2_Al^+^ and PO_3_HAl^+^ detected in ToF-SIMS are also shown (in red) with the monodentate mode for clarity purposes. The oxygen atoms in the native oxide are omitted for simplicity purposes.

The detection of PO^+^, PO_2_Al^+^ and PO_3_HAl^+^ from the FPA/Al before and after HW treatment is critical in understanding the interface chemistry or the interaction between the FPA anchoring group and the underneath native oxide layer of the Al substrate. Even though, from Figure 6, one can see that PO_2_Al^+^ is weaker than PO_3_HAl^+^, the relationships of their intensities to that of PO^+^ shown in Figure 5 are not explicit. By looking at their intensities, it is found that the intensities of PO_2_Al^+^ and PO_3_HAl^+^ are 1.5% and 12.0% of that of PO^+^. On the other hand, the resistance of the FPA/Al to HW attacking must be caused by the water-repelling nature of the FPA molecular chains in the SAMs that prevent water from penetrating to the underneath Al substrate. To shed light on these two important aspects of the FPA SAMs on an Al substrate, a monolayer of four FPA molecules self-assembled on the native oxide layer of an Al substrate is illustrated in Figure 7. Illustrated in the figure are binding modes between the anchoring group and the native oxide layer of Al, including the monodentate and bidentate with electrostatic configurations, which are regarded as the two most likely binding modes for SAMs of PAs formed on aluminum oxide [15,16].

The three ToF-SIMS detected positive ions related to the FPA anchoring group and its interaction with the Al substrate are denoted in the three monodentate configurations (Figure 7) for clarity purposes, where their fragmentations are indicated in red boxes. PO^+^ is likely due to the fragmentation of the phosphoryl group P(=O); however, contributions from the P-OH group or even the P-O-Al linkage could not be excluded. The detection of PO_2_Al^+^ and PO_3_HAl^+^ reflects the condensation reaction that covalently bonds the anchoring group to the native oxide layer of the Al substrate [6,16,26,41,46]. While PO_2_Al^+^ shows the covalent bonding between the phosphoryl group and an Al atom linked by an oxygen atom, PO_3_HAl^+^ represents the entire anchoring group and an Al atom. To our best knowledge, there has been no report on the detection of PO_2_Al^+^ and PO_3_HAl^+^. It is intriguing that the ToF-SIMS results could be explained reasonably well with the monodentate configuration. However, bidentate and tridentate configurations [6,15,16,46,47] cannot be excluded without comparisons of ToF-SIMS analyses of samples having known binding configurations. Also shown in Figure 7 is the binding mode of bidentate with electrostatic configuration, which is regarded as another possibility for SAMs of organo-PAs formed on aluminum oxide [15,16]. Fragmentation of PO_2_Al^+^ and PO_3_Hal^+^ from this binding mode is not impossible, which illustrates that interpretation of ToF-SIMS data without a reference is often challenging. Nevertheless, ToF-SIMS data also provide opportunities to explore the interface chemistry of SAMs formed on oxides [18]. For example, by comparing FPA/Al samples before and after annealing, differences in ion fragmentation patterns/trends might be detected, which is expected to be insightful in accessing the interface chemistry of SAMs and thus warrants a future investigation.

The chemical reaction of metallic Al and water [48] generates hydrogen gas, aluminum hydroxide and aluminum oxide [49,50]. When the temperature is raised to 90–100 °C, this chemical reaction will be accelerated, resulting in the observed nanostructured porous structures, as shown in Figure 2c and Figure 3c and as reported previously [26]. Therefore, the mechanism for HW-induced roughening simply requires the contact of water and the Al surface. When placed in (hot) water, the FPA SAMs serve to block the path for water to penetrate to the underneath Al substrate. It is worth noting that the strong covalent bonding between the FPA anchoring group and the native oxide layer of the Al substrate also contributes to preventing water from reaching the Al surface. It has been established that when the OPA anchoring group is weakly bonded (via H bonding) to a silicon oxide [18], water will find ways to disrupt the SAMs covering the substrate [12], despite the densely packed hydrocarbon chains of OPA repelling water. Therefore, the covalent bonding of FPA SAMs is also an important factor responsible for the SAM-provided protection for the underneath Al substrate against HW attack. The results discussed so far are for the FPA/Al sample immersed in HW for 90 s. We have confirmed that, even when the HW treatment time increased to 3 and 30 min, the measured static water CAs were 131.4° ± 0.7° and 131.3° ± 0.8° (with an example for each case shown in Figure S4), identical to that of the HW-treated FPA/Al for 90 s (Table 1), proving that the FPA SAMs protected the underneath Al substrate from HW attack for prolonged periods.

To demonstrate the feasibility of using FPA SAMs to pattern hydrophobic and hydrophilic areas on an Al substrate, we show in Figure S5a the steps to derivatize the unmasked portion of the Al substrate with FPA SAMs, followed by removing the mask and roughening the exposed area via HW treatment. Figure S5b,c show the differences in wettability between the hydrophilic and hydrophobic areas when the sample was subjected to mist generated from a humidifier and withdrawn from water, respectively. Though quite primitive, the results served as a proof of concept that FPA derivatization coupled with HW treatment is suitable in patterning hydrophobic and hydrophilic areas on an Al substrate. Future work is to make use of the existing microcontact printing technique [51] to create sophisticated wettability patterning on Al substrates with FPA solution as the ink [52,53].

## 4. Materials and Methods

The FPA (1*H*,1*H*,2*H*,2*H*-perfluoro-n-hexadecylphosphonic acid) used in this study was custom-made by Specific Polymers (Castries, France), which was identified as Proposal SP SSW 04 with a molecular formula of CF_3_(CF_2_)_13_(CH_2_)_2_P(=O)(OH)_2_. The FPA powder was heated to 100 °C to remove moisture before its 2 mm solution in a 1:1 mixture of methanol and chloroform or in trichloroethylene was prepared. The solution might be heated to 50–60 °C to make the FPA molecules completely dissolve in the solvent. Two Al films were coated on a Si wafer by radio frequency magnetron sputtering; the one used at Dali University had a thickness of 50 nm, while the one at the University of Western Ontario (UWO) was 15 nm. Coupons of a size of approximately 2 cm × 2 cm were cleaned with methanol in an ultrasonication bath for 15 min. Then, the coupons were immediately immersed in the FPA solution overnight to ensure a complete reaction between the FPA anchoring group and the underneath native oxide layer of Al. Finally, the FPA/Al samples were removed from the FPA solution, rinsed with methanol and dried with a nitrogen gas stream. No annealing was performed for the FPA SAMs formed on the native oxide layer of Al. For HW treatment, the samples were immersed for 90 s in ultrapure water at 90 °C in a beaker placed on a hot plate. An experiment on HW treatment for prolonged immersion times of 3 and 30 min was also carried out for wettability examination. For comparison purposes, OPA SAMs were also prepared on the 15 nm Al film under the same conditions as for the FPA SAMs preparation, except for the use of trichloroethylene as the solvent.

For the 50 nm Al film case, static water CAs on the bare Al and FPA/Al samples were measured with a goniometer (SDC-200, Sindin Precision Instrument, Dongguan, China) using a 3 μL droplet of ultrapure water (Eco-S15, Hitech Instruments, Shanghai, China). The ambient temperature and relative humidity were 18–25 °C and 30–60%, respectively. Static water CAs were measured in five spots on each sample and their averages and standard deviations were reported. For comparison with those of the samples made from the rougher 50 nm Al films, static CAs of a 3 μL droplet of ultrapure water (Synergy, Millipore, Molsheim, France) were measured at the UWO location on some of the FPA/Al samples made with the smooth 15 nm Al film using a drop shape analyzer (DSA30E, KRÜSS, Hamburg, Germany). The static water CAs were measured at the ambient temperature of 22 °C and the relative humidity of 25–30%.

The surface structures of the bare Al and FPA/Al samples upon HW treatment were examined using SEM (SU8023, Hitachi, Tokyo, Japan) at an accelerating voltage of 3 keV.

The dynamic force mode of an AFM (XE-100, Park Systems, Suwon, Republic of Korea) was used to study the surface morphology of the samples made with the 15 nm Al film using a cantilever (NSC15, MikroMasch, Sofia, Bulgaria) with a nominal spring constant of 40 N/m, resonant frequency of 325 kHz and tip radius of 10 nm. AFM images were obtained at 256 × 256 pixels.

The surface and interface chemistry of FPA/Al samples were investigated using an IONTOF TOF-SIMS IV (Münster, Germany) equipped with a BiMn liquid metal ion gun providing a pulsed, 25 keV Bi_3_^+^ cluster primary ion beam. This primary ion beam was used to bombard the sample surface to generate secondary ions, from which either positive or negative secondary ions, one polarity at a time, were extracted by an electric field, mass separated and detected via a reflectron-type time-of-fight analyzer, allowing parallel detection of ion fragments with a mass/charge ratio (*m*/*z*) up to ~900 within each cycle (100 μs) for the primary ion beam bombardment and the detection of the secondary ions. A pulsed, low-energy (~18 eV) electron flood was used to neutralize sample charging. The base pressure of the ToF-SIMS analytical chamber was 3 × 10^−7^ mbar. Positive and negative secondary ion mass spectra were initially calibrated using positive ions (e.g., C^+^, CH_3_^+^ and C_3_H_5_^+^) and negative ions (e.g., C^−^, CH^−^ and C_4_H^−^), respectively. If necessary, recalibration would be conducted using known ions. Spectra were collected on three spots at 128 × 128 pixels over an area of 400 μm × 400 μm. The mass resolutions for C_2_H^−^, PO_3_^−^, C_2_H_5_^+^ and C_3_H_5_^+^ were 4000, 5000, 4700 and 5300, respectively.

## 5. Conclusions

We have shown that FPA SAMs formed on the native oxide of Al protected the underneath Al substrate from HW attack, as evidenced by the intact surface morphology and wettability, as well as surface and interface chemistry. ToF-SIMS analyses further revealed an ion (PO_3_HAl^+^) diagnostic of the condensation reaction between the FPA molecules and the Al substrate, which is important in understanding the interface chemistry of the SAMs. The strong bonding of the anchoring group of FPA on the native oxide of Al and the hydrophobic nature of the perfluorocarbon tails are expected to lead to the development of pattering hydrophobic and hydrophilic areas on an Al substrate assisted by HW treatment for creating functional surfaces for applications in harvesting water from atmospheric moisture.

## Figures and Tables

**Table 1:** Static water contact angles (CAs) for bare Al and FPA/Al before and after HW treatment at 90 °C for 90 s.

Sample	Averaged CA (°)
Cleaned Al	45.9 ± 1.3
FPA/Al	134.4 ± 1.4
HW-treated Al	9.0 ± 0.5
HW-treated FPA/Al	131.1 ± 0.1

## Data Availability

Data are available upon reasonable request.

## Supplementary Materials

The following supporting information can be downloaded at: https://www.mdpi.com/article/10.3390/molecules29030706/s1, Figure S1: An AFM image of the 50 nm Al film; Figure S2: An example of a CA measured on the FPA/Al sample (prepared on the 15 nm Al film) before and after HW treatment for 90 s; Figure S3: An example of a CA measured on the OPA/Al sample (prepared on the 15 nm Al film) before and after HW treatment for 90 s; Figure S4: An example of a CA measured on the FPA/Al sample (prepared on the 50 nm Al film) subjected to HW treatment for 3 and 30 min; Figure S5: A hydrophilicity and hydrophobicity patterning trial using FPA SAMs and HW treatment and its wettability tests.
