# Supplementary material for: Self-Assembled Monolayers of a Fluorinated Phosphonic Acid as a Protective Coating on Aluminum
Source: Molecules. 2024 Feb 3;29(3):706. doi: 10.3390/molecules29030706 (PMC10856205; doi:10.3390/molecules29030706)
Supplement: Supplementary file 1 [file molecules-29-00706-s001.zip › molecules-2829251-supplementary.pdf]

Supplementary Materials

Self-Assembled Monolayers of a Fluorinated Phosphonic Acid as a Protective Coating on Aluminum

Zhuoqi Duan [1], Zaixin Xie [1], Yongmao Hu [1], Jiawen Xu [2,3], Jun Ren [2,3], Yu Liu [3] and Heng-Yong Nie [2,4]

[1] College of Engineering, Dali University, Dali 671003, China

[2] Surface Science Western, The University of Western Ontario, London, Ontario, N6G 0J3, Canada

[3] School of Mechanical Engineering, Jiangnan University, Wuxi 214122, China

[4] Department of Physics and Astronomy, The University of Western Ontario, London, Ontario, N6A 3K7, Canada

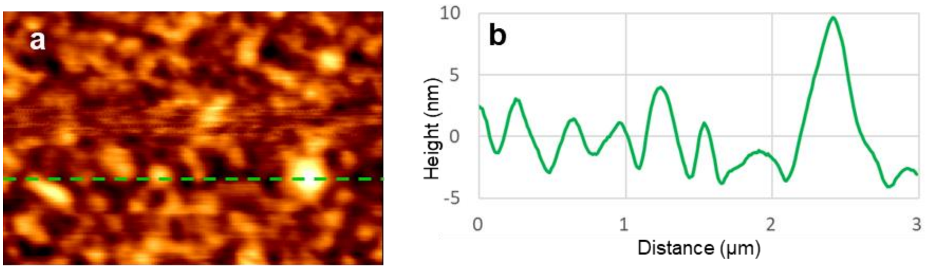

Figure S1. Topographic image (a) obtained on the 50-nm Al film using atomic force microscopy and a profile (b) isolated from the image (indicated by the inserted broken line). The scan area of the image is 3 µm × 2 µm and the height range is 15 nm.

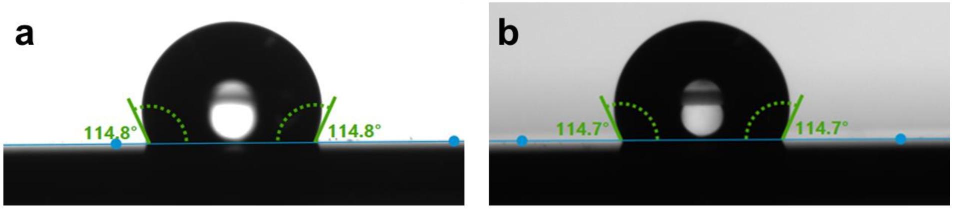

Figure S2. Photographs of a 3-µl ultrapure water droplet placed on FPA/Al before (a) and after (b) HW treatment, showing typical CAs of 114.8° and 114.7°, respectively. The temperature of HW was 90 °C and the thickness of the Al film used was 15 nm.

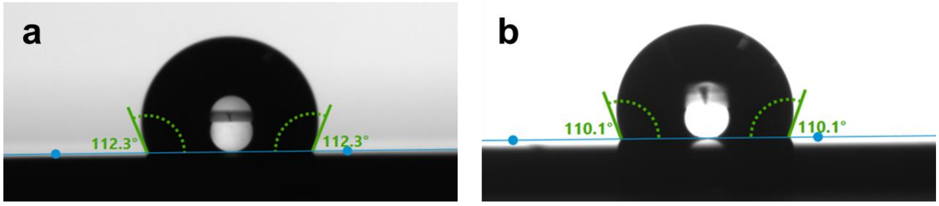

Figure S3. Photographs of a 3-μl ultrapure water droplet placed on OPA/Al before (a) and after (b) HW treatment, showing typical CAs of 112.3° and 110.1°, respectively. The temperature of HW was 90 °C and the thickness of the Al film used was 15 nm.

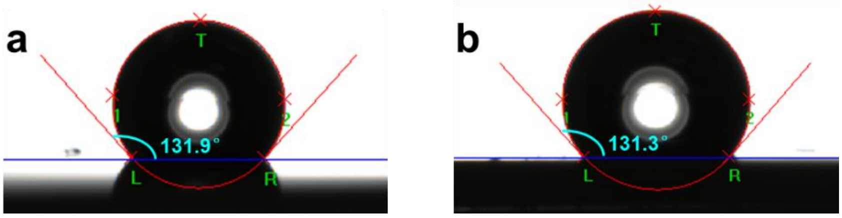

Figure S4. Photographs of a 3-μl ultrapure water droplet placed on HW-treated FPA/Al for (a) 3 min and (b) 30 min, showing CAs of 131.9° and 131.1°, respectively. The temperature of HW was 90 °C and the thickness of the Al film used was 50 nm.

To demonstrate the feasibility of using FPA-derivatization to pattern hydrophobic and hydrophilic areas on an Al surface, we show in Figure S5a the steps to derivatize the unmasked portion of the Al surface with FPA SAMs, followed by removing the mask, an Al foil taped down using an adhesive tape and immersing the sample to HW to roughen the unprotected Al area. The final product is a hydrophilic area with the rest of the sample being hydrophobic. Shown in Figure S5b is a photograph illustrating differences in wettability between the hydrophilic and hydrophobic areas. Water wetted the hydrophilic area well so that it was invisible to the eye (except for a few imperfect spots). By contrast, water beaded up on the hydrophobic (i.e., FPA-derivatized) area. The accumulation of water wetting the hydrophilic area appeared as larger droplets at the hydrophilic-hydrophobic boundary. Figure S5c shows the wet hydrophilic area and dry hydrophobic area after the sample was withdrawn from water, with wetted hydrophilic area and dry hydrophobic area being wetted and the hydrophobic area (they may appear the same because the water film covering the hydrophilic area is transparent). The water accumulated at the boundary of the two areas (Figure S2c) is due to the fall of water from the wet hydrophilic area but blocked by the water-repelling hydrophobic area.

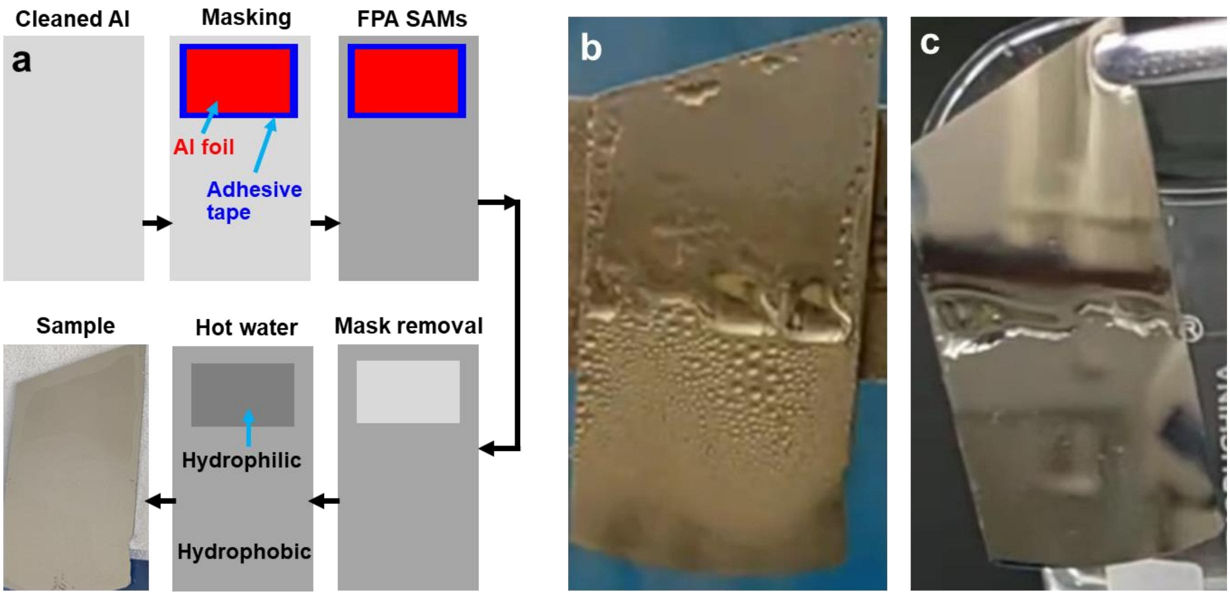

Figure S5. The steps to (a) pattern hydrophilic and hydrophobic areas on an Al film deposited on a Si wafer via FPA derivatization and HW treatment, and a wettability test of (b) using the mist generated from a humidifier and (c) withdrawing from water. The temperature of the HW was 90 °C and the thickness of the Al film deposited on a Si wafer was 50 nm.
